# A Rare Case of Nonsecretory Multiple Myeloma in Lagos, Nigeria: A Case Report and Literature Review

**DOI:** 10.1155/2015/648069

**Published:** 2015-11-15

**Authors:** Ebele Uche, Akinsegun Akinbami, Sarah John-Olabode, Adedoyin Dosunmu, Majeed Odesanya

**Affiliations:** ^1^Department of Haematology and Blood Transfusion, Lagos State University College of Medicine, Ikeja, Nigeria; ^2^Department of Haematology and Blood Transfusion, College of Medicine, University of Lagos, Idi Araba, Lagos State, Nigeria; ^3^School of Life and Health Sciences, Aston University, Birmingham, UK

## Abstract

Multiple myeloma (MM) is a plasma cell disorder associated with clonal proliferation of plasma cells. Nonsecretory multiple myeloma (NSMM) is a rare variant of MM and accounts for approximately 1% to 5% of all cases. It is defined as symptomatic myeloma without detectable monoclonal immunoglobulin on serum or urine electrophoresis. This variant usually poses a diagnostic challenge to the clinician. We present a 60-year-old Nigerian man who was investigated extensively for bone pain, weight loss, and anaemia. He was eventually diagnosed as having nonsecretory multiple myeloma based on histology and immunohistochemistry results of bone marrow trephine biopsy. He is currently being managed with bortezomib, doxorubicin, and thalidomide, as well as zoledronic acid. He is also on anticoagulation. He continues to show remarkable clinical improvement. We describe this case report and literature review for better awareness amongst medical practitioners and pathologists.

## 1. Introduction

MM is a disease characterized by the presence of clonal plasma cells in the marrow. These malignant plasma cells secrete an abnormal immunoglobulin causing a monoclonal gammopathy that can be identified in the serum and/or urine by electrophoresis. The disease is characterized by end organ damage; manifestations of which include hematologic, renal, or bone complications [1]. Thus, the patient with MM can present with anaemia, hypercalcaemia, lytic bone lesions and renal failure amongst others.

MM accounts for 2% of all cancer deaths and nearly 20% of deaths caused by haemtological malignancies in the United States. The incidence is twofold higher in African Americans than in Caucasians, with a significantly higher incidence in males [2].

MM and NSMM have essentially the same clinical and radiologic features. However, in the case of NSSM, the plasma cells fail to secrete an immunoglobulin and therefore both serum and urine electrophoresis are normal [3]. Diagnosis depends on bone marrow biopsy and demonstration of plasmocytes by immunohistochemistry. The first case of this was described in 1958 and the reported incidence has ranged from 1% to 5% of all cases of MM [3].

## 2. Case Report

**Figure 1:**
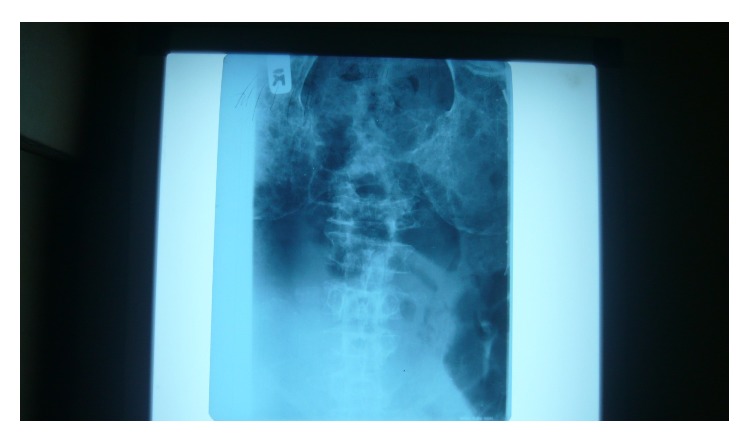


A 60-year-old male was referred to our clinic with 5-month history of gradual but progressive weight loss, recurrent fever, anorexia, progressive fatigue, and generalized bone pains. Two months after the onset of these symptoms, he developed inability to walk. There was no associated sphincteric dysfunction or sensory loss. There was no history of chronic cough. At the time of presentation, significant examination findings were pallor, inability to walk, and general bone tenderness. X rays of the skull, thoracolumbar spine, and pelvis showed lytic lesions (Figure 1). Blood film and bone marrow aspiration were essentially normal with <5% plasma cells reported in the bone marrow. Erythrocyte sedimentation rate, C reactive protein, and serum protein electrophoresis were normal and urinary Bence Jones protein was negative. Laboratory data are summarized in Table 1.

A bone marrow trephine biopsy was done and showed osteosclerotic changes and focal scalloping with prominent osteoclastic activity. The marrow was moderately to markedly hypercellular and was densely infiltrated by a cellular infiltrate. The cells were mostly small cells with fairly condensed chromatin. They showed a variable amount of cytoplasm and there was a fair sized subpopulation of plasmacytoid lymphocytes.

Immunohistochemistry showed positivity of CD138 (which strongly supports the diagnosis of plasma cell myeloma) and cyclin D1 (which is associated with cases of plasma cell myeloma with lymphoplasmacytic morphology and correlates with the presence of the translocation t(11;14)(q13;q32)).

## 3. Discussion

MM is a disorder of the bone marrow and accounts for one to two percent of all malignancies. In Lagos, Nigeria, Akinbami et al. reported 12.2% prevalence in all adult hematooncology cases [4].

NSMM is a rare variant characterized by the absence of detectable M protein in serum and urine. It was first described in 1958 and a retrospective study of 869 cases of MM conducted in 1975 suggested that the prevalence of NSMM was 1% [5]. Since then, there have been several case reports describing this variant of multiple myeloma [5–7].

Two distinct types of NSMM have been described. In the first type, the plasma cells produce immunoglobulins but are unable to secrete it out of the cell, possibly due to reduced permeability or absence and alteration of intracellular light chains. This form of NSMM is known as the “producer” type or true NSMM. The second is the nonproducer type, where plasma cells are unable to produce immunoglobulin [8–10].

There have been several hypotheses to explain the absence of demonstrable M protein in serum and urine of patients with NSMM. One theory postulates that the producer type may be as a result of increased breakdown of abnormal immunoglobulin produced, while the nonproducer type may be as a result of problems with the assembly process of proteins, thereby leading to difficulty with immunoglobulin heavy and light chain synthesis [10–12].

The diagnosis of MM is based on the demonstration of bone marrow plasmacytosis of =10%, monoclonal protein in serum and/or urine, and myeloma related organ dysfunction (hypercalcaemia, renal insufficiency, anaemia, and lytic bone lesions) [13].

Due to the absence of M protein in serum and urine, our patient did not meet the criteria for the diagnosis of MM. He also failed to meet the criteria for the diagnosis of NSMM, which according to the International Myeloma Working Group is defined by the absence of M protein in serum or urine, bone marrow plasmacytosis, and Related Organ or Tissue Impairment (ROTI) [13].

However, about 5–10% of symptomatic patients may have less than >10% of plasmacytosis, as was the case in this patient [14]. In such instances, diagnosis may be made with tissue biopsy and histology, presence of lytic bone lesions, and raised serum levels of *β*-2M [13].

Once diagnosed, the treatment of NSMM remains the same as for multiple myeloma. Recent advances in pharmacologic treatment include use of thalidomide, lenalidomide, and bortezomib as active agents in MM. Due to the increased significant risk of thromboembolic events associated with the use of these immune modulators, anticoagulation is frequently incorporated in management.

Induction chemotherapy followed by autologous stem cell support is widely used as treatment in patients with multiple myeloma.

However, in our environment, facilities for stem cell transplantation are limited and therefore treatment is mostly reliant on the use of chemotherapy. Also due to the patient's age, he may not be fit for autologous stem cell transplant. We hope to refer the patient for assessment for transplant after completion of induction chemotherapy.

In conclusion, nondemonstration of an M protein in either serum and/or urine does not negate a diagnosis of MM. NSMM should be suspected in patients with clinical features of MM but with no monoclonal gammopathy. Bone marrow aspiration and trephine biopsy in addition to more advanced techniques like immunohistochemistry should always be considered.

## Figures and Tables

**Table 1:** Laboratory results.

Parameter	Reference range	Case
Hemoglobin^*∗*^	14–18 g/dL	8.3 g/dL
ESR	15 mm/hr	11 mm/hr
Calcium	2–2.55 mmol/L	2.33 mmol/L
Urea	2.5–6.4 mmol/L	5.6 mmol/L
Creatinine	53–115 *µ*mol/L	71 *µ*mol/L
Albumin	30–50 mg/dL	29 mg/dL
C reactive protein	0–10 mg/dL	2 mg/dL
Lactate dehydrogenase	91–108	106
Bence Jones protein	Absent	Absent
Urine electrophoresis	Normal	Normal
Alpha 1 globulin	2–6 g/L	4 g/L
Alpha 2 globulin	3–10 g/L	9 g/L
Beta 1 globulin	3–6 g/L	3 g/L
Beta 2 globulin	2–6 g/L	3 g/L
Gamma globulin	6–15 g/L	7 g/L
M-component	0 g/L	0 g/L
Β2 microglobulin^*∗*^	<2.4 mg/L	5.1 mg/L

^*∗*^Deranged parameter.
